# Granulomatous mastitis secondary to *Corynebacterium* requiring surgical intervention: a complicated diagnosis

**DOI:** 10.1093/jscr/rjad211

**Published:** 2023-04-22

**Authors:** Charles Lu, Vincent Marcucci, Erica Kibbe, Roshani Patel

**Affiliations:** Department of Surgery, Jersey Shore University Medical Center, Neptune, NJ, USA; Department of Surgery, Jersey Shore University Medical Center, Neptune, NJ, USA; Department of Surgery, Jersey Shore University Medical Center, Neptune, NJ, USA; Department of Surgery, Jersey Shore University Medical Center, Neptune, NJ, USA

## Abstract

*Corynebacterium* species is a Gram-positive bacillus endogenous to human integument that has previously been associated with idiopathic granulomatous mastitis. The diagnosis and treatment of this bacteria may be complicated by inability to distinguish colonization from contamination and infection. We present an uncommon case of granulomatous mastitis associated with negative wound cultures requiring surgical intervention.

## INTRODUCTION

Granulomatous mastitis is an uncommon inflammatory breast condition that has a controversial etiology and is usually observed in parous women of childbearing age. Initially granulomatous mastitis was thought to be idiopathic in etiology or postulated as an autoimmune response, but recent literature exposes alternate infectious causes. It is known that tuberculosis and fungal infections can lead to this condition; however, *Corynebacterium* is noted to be an emerging pathogen. This condition typically presents with a unilateral breast mass with varying degrees of tenderness that can form an abscess with granulomatous inflammation and can have complications of both sinus tracts and fistulas. As *Corynebacterium* is a known skin flora, it can be misinterpreted as a contaminant instead of a pathogenic etiology. The presence of positive cultures on a sterile aspirate culture lends to exposing *Corynebacterium* as a novel pathogen. This case report describes a parous female who presented with a unilateral breast mass. The mass was found to be caused bya granulomatous mastitis due to *Corynebacterium*.

## CASE PRESENTATION

A 40-year-old female initially presented to a breast surgery clinic due to concern for a breast lump after a traumatic event. She subsequently underwent screening mammography, which was abnormal and concerning for malignancy. She ultimately underwent core needle biopsy which revealed granulomatous mastitis. She was treated with a 2-week course of Keflex, resulting in no improvement. She was then placed on a prednisone taper with transient symptomatic improvement. Prior to referral to our breast surgery team, an oral course of doxycycline was completed with persistence of her symptoms.

**Figure 1:**
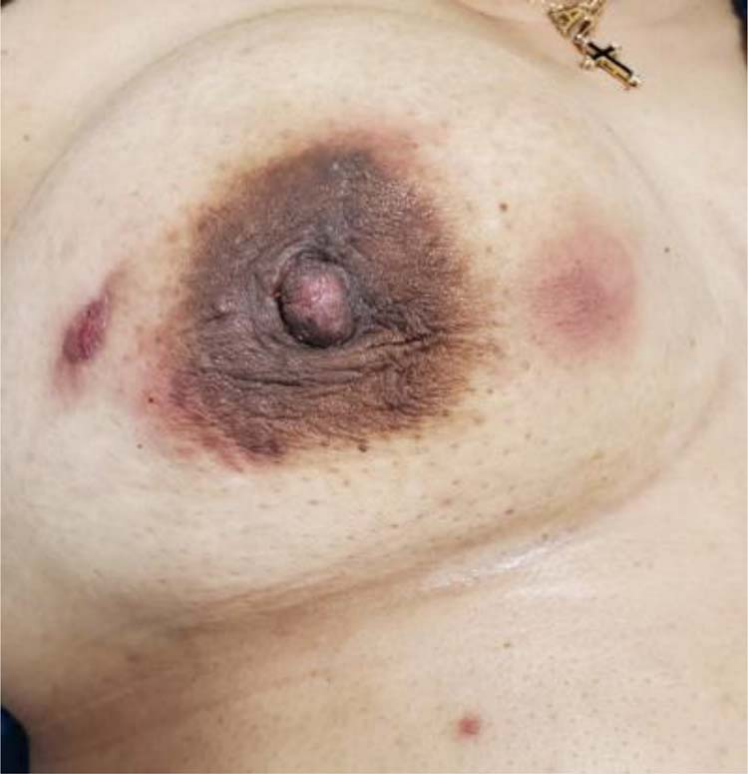
Gross image demonstrating right breast with erythematous areas corresponding to abscess collections at the 3:00 position 4–5 cm from the nipple, 12:00 areolar border and 9:00, 3–4 cm from the nipple, with an apparent fistulous connection.

**Figure 2:**
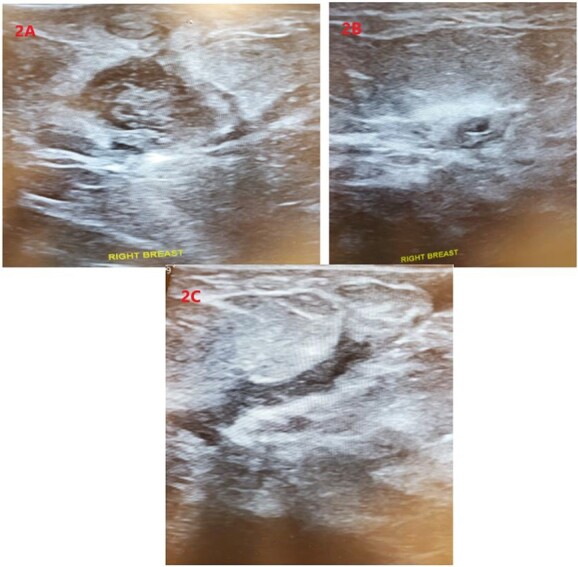
Sonographic imaging of the right breast identifying two complex fluid collections (**A**) and (**B**) at the 3:00 position. (**C**) Showing a fistula tract.

Upon physical examination, the patient was found to have multiple red, indurated and tender areas on her right breast with concerns for a fistula to the lateral aspect of her nipple as demonstrated in Fig. 1. A sonogram was performed in the office identifying multiple abscess cavities with confirmation of a fistulous tract as shown in Fig. 2A–C. The patient had two aspiration procedures of her fluid collections with the initial set of wound cultures that were not successful in isolating an organism. Her infection did not resolve with further penicillin administration. Concerns for multidrug-resistant organisms and acid fast bacilli were included in the differential diagnosis.

With progression of her symptoms for over a year and lack of a diagnostic organism with poor response to medical management, the decision was made to take the patient to the operating room for incision/drainage and debridement with partial closure and packing. Operative findings revealed significant granulation tissue and multiple abscesses as previously described with a fistula to the skin. Perioperative antibiotics included vancomycin and a gentamicin/clindamycin irrigant solution to wash out the abscess cavities. Surgical debridement, however, did not yield cultures. She then developed a new collection within 1 month postoperatively requiring aspiration; cultures at this time grew *Corynebacterium kroppenstedtii*. It was believed that the cultures that ultimately grew *Corynebacterium* may have been due to a stricter set of protocols used to isolate the bacteria. Of note, the patient did have a follow-up aspiration while on antibiotics which did not grow any further organisms.

She began a 6-week course of doxycycline, taking 100 mg po twice daily. The patient continued to follow up in the office for wound checks. Nearing the end of her 6-week treatment course with doxycycline, the patient’s recovery was complicated by a small collection at the 1 o’clock position on the right breast. Due to concerns for resistant bacterium, the collection was subsequently drained and central access was obtained for initiation of a prolonged course of IV vancomycin. However, during the first dose of IV vancomycin, the patient developed signs of vancomycin flushing syndrome and was subsequently transitioned to ceftriaxone. The patient continued to have recurrent collections while on ceftriaxone requiring further operative drainage procedures. Ultimately, her clinical condition improved with no new collections or symptoms 5 months after her last drainage procedure. 

## DISCUSSION

Idiopathic granulomatous mastitis (IGM) was first described in 1972, and since then, there has yet been a definitive approach to identify and treat the disease. Although rare, this condition is typically observed more frequently in women of childbearing age and with a higher incidence in Hispanic, Native American, Middle East and African descent [1]. GM frequently mimics malignancy of the breast and thus, invariably places patients at the center of diagnostic uncertainty [2, 3]. The pathogenesis of IGM remains largely unknown although a progression from subclinical mastitis to mastitis and finally abscess development with granuloma formation has been suggested [3, 4]. There has also been a reported 15% of patients that presented with lymphedema [5]. There are many competing theories on the etiology of granulomatous mastitis including autoimmune, hypersensitive or infectious [6]. There is limited literature demonstrating an association between infection with *Corynebacterium* and IGM. Aspiration can often fail to produce the organism due to the commonly identified centrally located necrotic tissue [4]. When cultured, these organisms also require a longer incubation period on special media since corynebacterial survive in lipid-filled vacuoles [7, 8]. This is important when deciding an appropriate treatment course as many antimicrobials are hydrophilic [9, 10]. Additionally, instrumentation or aspiration may predispose individuals to develop new collections that may harbor *Corynebacterium* species due to a possible inflammatory response to epithelial damage [11]. Optimal treatment is certainly controversial and may consist of a trial of antibiotics, steroids or immunomodulators such as methotrexate in combination with possible surgery [12]. Although corticosteroids or other immunosuppressive agents may slow the development of granulomatous disease, further research is warranted [8, 12]. Ultimately, surgical management has showed the highest remission rate [8].

## CONCLUSION

The primary goal of any patient that presents with a new onset breast mass is early and accurate diagnosis. Granulomatous mastitis continues to present a challenge for accurate diagnosis and may subject patients to a prolonged clinical course. The need for new approaches to identify the underlying cause of this disease is warranted to prevent delayed diagnosis and unwarranted medical management.
